# Leukocyte Activity Is Altered in a Ground Based Murine Model of Microgravity and Proton Radiation Exposure

**DOI:** 10.1371/journal.pone.0071757

**Published:** 2013-08-14

**Authors:** Jenine K. Sanzari, Ana L. Romero-Weaver, Gabrielle James, Gabriel Krigsfeld, Liyong Lin, Eric S. Diffenderfer, Ann R. Kennedy

**Affiliations:** Department of Radiation Oncology, University of Pennsylvania Perelman School of Medicine, Philadelphia, Pennsylvania, United States of America; King’s College London School of Medicine, United Kingdom

## Abstract

Immune system adaptation during spaceflight is a concern in space medicine. Decreased circulating leukocytes observed during and after space flight infer suppressed immune responses and susceptibility to infection. The microgravity aspect of the space environment has been simulated on Earth to study adverse biological effects in astronauts. In this report, the hindlimb unloading (HU) model was employed to investigate the combined effects of solar particle event-like proton radiation and simulated microgravity on immune cell parameters including lymphocyte subtype populations and activity. Lymphocytes are a type of white blood cell critical for adaptive immune responses and T lymphocytes are regulators of cell-mediated immunity, controlling the entire immune response. Mice were suspended prior to and after proton radiation exposure (2 Gy dose) and total leukocyte numbers and splenic lymphocyte functionality were evaluated on days 4 or 21 after combined HU and radiation exposure. Total white blood cell (WBC), lymphocyte, neutrophil, and monocyte counts are reduced by approximately 65%, 70%, 55%, and 70%, respectively, compared to the non-treated control group at 4 days after combined exposure. Splenic lymphocyte subpopulations are altered at both time points investigated. At 21 days post-exposure to combined HU and proton radiation, T cell activation and proliferation were assessed in isolated lymphocytes. Cell surface expression of the Early Activation Marker, CD69, is decreased by 30% in the combined treatment group, compared to the non-treated control group and cell proliferation was suppressed by approximately 50%, compared to the non-treated control group. These findings reveal that the combined stressors (HU and proton radiation exposure) result in decreased leukocyte numbers and function, which could contribute to immune system dysfunction in crew members. This investigation is one of the first to report on combined proton radiation and simulated microgravity effects on hematopoietic, specifically immune cells.

## Introduction

It is now well established that spaceflight alters immune function [1]–[6] by mechanisms which are poorly understood. The changes in the immunological parameters studied thus far occur within a few days of exposure to the space environment. Factors in the space environment contributing to immune dysregulation during and post-spaceflight include exposure to microgravity, stress, deconditioning (reduced physical activity and shift of fluids), and radiation. Primary immune defense heavily relies on immune cell distribution and function, and is clearly influenced by a combination or synergy of any of the factors described above that exist in the space environment.

Of the blood cell types, lymphocytes are the most sensitive to ionizing radiation exposure. T cells, or T lymphocytes, processed in the thymus, secrete lymphokines, which orchestrate signaling to lymphocytes and other immune cells to promote cell activation, proliferation, destroy target cells, and incite macrophages. Blood lymphocytes isolated from astronauts upon re-entry after a prolonged spaceflight exhibit decreased responses in mitogen reactivity, T-lymphocyte proliferation, and IL-2 production [7]. Space laboratory studies using rat splenocytes indicate dramatic shifts in T lymphocyte subsets as well as decreased cell division in bone marrow cells post-flight [8]. A follow up study using bone marrow cells isolated from rhesus monkeys upon landing indicated decreased cytokine production and cytokine receptor expression [9]. The functionality of lymphocytes is compromised due to space flight. The activation of lymphocytes is also altered. When human lymphocytes are exposed to microgravity in a rapidly rotating clinostat, Concanavalin A (ConA) stimulated T cell activation is depressed [10], [11]. During flight, the activation of cultured human lymphocytes is depressed to less than 3% of the ground controls when exposed to ConA [12]. Microarray analysis of T lymphocytes exposed to mitogen in a vectorless gravity environment (using a random positioning machine) revealed that early T cell activation-associated gene expression is in fact, suppressed [13].

Hematological and immune system effects from ionizing radiation exposure (similar to the expected or estimated spaceflight doses) are established. Previous data indicate a dose-dependent decrease in peripheral blood cell counts, particularly lymphocytes, after whole body proton radiation exposure [14]–[16]. Bone marrow derived lymphocyte numbers are also decreased after whole body proton radiation exposure [17]. Limited studies have been reported on the function and activation of lymphocytes after proton radiation exposure [18], and there are no available reports on lymphocyte function and activation in response to ground-based microgravity simulation with combined proton radiation exposure.

Reports on the combined effects of ionizing radiation and simulated microgravity exposure on parameters associated with the immune response are extremely limited. Utilizing a rotating cell culture system, early studies reported that microgravity simulation inhibited gamma radiation-induced programmed cell death in either cultured peripheral blood lymphocytes [19] or lymphoblastoid cells [20], compared to cells maintained at normal gravity. A more recent report identified miRNAs and miRNA-mediated DNA-damage response genes that are not responsive to gamma-ray radiation exposure after simulated microgravity exposure, suggesting that the identified miRNAs may be responsible for the differential cell fate of irradiated cells exposed to microgravity or not [21]. These experiments contribute greatly to the potential risks associated with exposure to the space environment.

Although it is impossible to simulate true microgravity on Earth for mammalian systems, i.e., animal model systems, ground-based models have been developed, including head-down bed rest, water immersion, and harness restraint. The most widely accepted rodent model for simulating microgravity, the 30° antiorthostatic suspension technique, also known as the hindlimb unloading (HU) model, has been reviewed extensively and applied as a model to study infection, renal function, muscle atrophy, cardiovascular deconditioning, and bone homeostasis [22]–[26]. Using this model, a decrease in mouse spleen and thymus lymphocytes via apoptosis is observed after 2 days of HU, and this HU-induced reduction of lymphocytes was blocked by endogenous opioids and corticosteroids [27]. In a separate study in which rats were exposed to the HU system for 16 days, a decrease in thymocytes was observed and actually reversed with Substance P treatment [28].

In this report, the HU model was employed to investigate the combined effects of proton radiation and simulated microgravity on immune cell parameters including lymphocyte subtype populations and lymphocyte activity.

## Methodology

### Animals

Female ICR (Imprinting Control Region) mice (Taconic Farms, Inc., Hudson, NY, USA) 6–8 weeks of age were group-housed in standard laboratory vivarium caging with *ad libitum* access to both food and water. All procedures for the animal care and treatment were approved by the Institutional Animal Care and Use Committee of the University of Pennsylvania.

Animals were randomly assigned to treatment groups. After 1 week of acclimation to the animal facility, animals were singly housed in the custom-built HU cages for 3 days. The groups of animals that were to be suspended (+/− radiation) were placed in suspension 2 days prior to the start of the experiment (2 days prior to irradiation or sham-irradiation exposure). The sample size of each treatment group were as follows for the 4 day and 21 day time points, respectively: No treatment group = 6, 6; 2 Gy group = 6, 5; HU group = 4, 4; 2 Gy + HU group = 5, 5.

The HU mice were prepared for suspension by placing a Steri-Strip™ (3M, St. Paul, MN) at the base of the tail. The adjustable bead chain was placed parallel to the tail and taped with athletic tape. The hindlimbs of the mouse were then elevated at a 30° angle [22] from the floor of the cage by placing the adjustable bead chain in an end coupling that was allowed to freely move across a rod at the top of the cage. All mice (suspended and non-suspended) were provided food and water *ad libitum*. The non-suspended controls were pair-fed according to the previous day’s average consumption of the suspended mice.

### Radiation

After 3 days of acclimation to the caging, non-anesthetized animals were restrained in custom designed Plexiglass chambers and exposed to a single whole body proton radiation dose of 2 Gy, delivered at a dose rate of 0.5 Gy/min or were sham-irradiated on day 0. The mice were irradiated at the Roberts Proton Therapy Center at the University of Pennsylvania. The proton beam was produced by an IBA cyclotron system. The 230 MeV proton beam extracted from the cyclotron was degraded using the energy selection system to a nominal energy of 151 MeV or range of 16 cm water equivalent thickness (WET, IBA Proton Therapy System Maintenance Manual for the Roberts Proton Therapy Center). The degraded beam was delivered in double scattering mode with a uniform spread out Bragg peak (SOBP) modulation width of 5 cm using a horizontal beam with gantry angle 270°. Eight mouse enclosures with wall thickness of 1.6 mm and dimensions of 4.1 cm (depth)×4.1 cm (height)×7.2 cm (width) were arranged in a 4−2 array forming a 16.4 cm×14.2 cm target area. A 23 cm×17 cm opening in the tungsten multi-leaf collimator (MLC) shaped the beam. Under such conditions, the dose was larger than 95% of the flat region and penumbra regions were removed from the 20.6 cm×17 cm area usable for animal irradiation at the gantry isocenter. The center of the enclosure array was placed at the gantry isocenter with an additional 11 cm WET of solid water plastic (Gammex Inc., Middleton, WI, USA) placed directly in front of the array further degrading the proton beam energy to approximately 78.4 MeV or a range of 5 cm WET. 5 cm WET of solid water plastic was placed directly behind the enclosure array. The mouse enclosures were irradiated with a range of proton energies forming the uniformly modulated dose region of the SOBP. The dose averaged linear energy transfer (LET) of the proton radiation is low (<10 keV/µm) within the mid-SOBP where the mice are located and rises to higher LET (>10 keV/µm) towards the downstream edge of the SOBP, which lies beyond the mouse enclosures. Dosimetry verification was performed before the irradiations with a 2D ion chamber array (I’m*RT* MatriXX from IBA dosimetry, Schwarzenbruck, Germany) placed at the middle of SOBP. Immediately following the irradiation exposure, mice were returned to their respective home cages and were placed back in suspension (as described above) or for the non-suspended controls, were simply placed in their HU cage without suspension.

### Complete Blood Cell Counts

The designated experimental endpoints were evaluated at 4 days and 21 days. Previously, we have shown that blood cell counts vary within the first 48 hours of exposure to HU, after which time the counts are not different in a statistically significant manner, compared to the non-suspended controls. The changes in blood cell counts is presumably attributed to stress of the HU system [29]. To study an “acute” time point after radiation +/− HU treatment, a 4 day time point was chosen so that the initial stress response to the suspension system would be minimized. Given the technical difficulties in keeping mice suspended for long periods of time, the 21 day time point was manageable to study “long term” effects of white blood cells. Most of the white blood cells generally survive several days, whereas the monocytes and lymphocytes survive for weeks or longer; therefore, the 21 day time point was suitable to study the effects of the treatment(s) on the white blood cells with the longer survival time.

At the completion of each study, animals were euthanized by carbon dioxide asphyxiation. Cardiac blood was collected and the samples were sent to Antech Diagnostics (Lake Success, NY) and a complete blood cell (CBC) count with differential was performed using the Cell-Dyn 3500 within 24 hours of blood collection. Blood cell counts are expressed as a fraction of the control. For example, the white blood cell count of the HU group/the average white blood cell count of the No treatment group = fraction of control.

### Flow Cytometry

Spleens were excised at the time of euthanasia and prepared for flow cytometry. Briefly, a single cell suspension was prepared by teasing the spleen apart with the plunger of a syringe in a petri dish containing staining buffer (eBiosciences, San Diego, CA, USA). The cell suspension was passed through a cell strainer to remove debris. Total spleen cells were collected by centrifugation and erythrocytes were lysed using commercially available lysis buffer (eBiosciences). The lysis buffer was neutralized with the addition of phosphate buffered saline and leukocytes were counted using a Z Series Coulter Counter (Beckman Coulter, Inc., Brea, CA, USA).

Cells were washed with staining buffer (eBiosciences), and incubated with purified anti-mouse CD16/32 to block non-specific Fc receptor binding. Three separate tubes were prepared for cell surface phenotyping. The first tube was a cocktail of anti-CD3 (phycoerythrin [PE]-conjugated), anti-CD4 (allophycocyanin [APC]-conjugated), and anti-CD8 (flourescein [FITC]-conjugated). The second tube was a cocktail of anti-CD3 (PE-conjugated) and anti-CD19 (FITC-conjugated). All antibodies were purchased from eBiosciences. The last tube contained propidium iodide (Sigma-Aldrich, St. Louis, MO, USA) to discriminate viable vs. dead cells. Cells were stained in the dark at 4°C for 30 min. with subsequent washing with staining buffer. Cells were analyzed on a FACSCalibur (BD Biosciences, San Jose, CA, USA) with CellQuest Pro software (BD Biosciences) and further analyzed using FlowJo Analysis Software (Tree Star Inc., Ashland, OR, USA). 10,000 events were collected.

### Splenic T Lymphocyte Isolation and Activation

Approximately 1×10^7^ leukocytes, isolated from the spleen of each animal, were used for the isolation of T lymphocytes. A mixture of monoclonal antibodies against non-T cells were added to each sample. Dynabeads (Invitrogen, Carlsbad, CA) were added to the samples and bound to the non-T cells. The bead bound cells were separated by a magnet and discarded. The remaining T cells were counted and 10^6^ cells from each group were dispensed to a microplate. All samples were activated using the activation cocktail of CD3/CD28/CD127 and recombinant IL2. Cells were cultured in a humidified CO_2_ incubator for 24 hours. After 24 hours, cells were prepared for surface detection of CD69, the Early Activation Marker, by flow cytometry (see method above). The anti-CD69 (peridinin chlorophyll 5.5 [PerCP-Cy5.5]-conjugated) antibody was obtained from eBiosciences.

### Cell Proliferation Assay

The Vybrant MTT Cell Proliferation Assay Kit (Molecular Probes, Eugene, OR) was used to determine the cell number after exogenous activation. Cells were seeded in a microplate containing the activation cocktail. A negative control was included containing fresh media with no activation cocktail. The MTT stock solution was added to each well and the microplate was placed in a humidified CO_2_ incubator for 4 hours. The MTT in each well is converted to formazan, which is solubilized by the addition of sodium dodecyl sulfate in diluted hydrochloric acid. The absorbance was read at 570 nm.

### Statistical Analyses

The data obtained from the flow cytometry, proliferation, and the CBC assays were all analyzed by one-way ANOVA. Comparisons between each treatment group were made using Tukey’s test after one-way ANOVA analysis. The statistical software used was Graphpad version 5.0. The histograms represent average cell population, cell count (expressed as a fraction of the control or No treatment group), or proliferation index +/− standard deviation, with n = 4–6 animals per treatment group. Changes between groups were considered statistically significant at p<0.05.

## Results

### Blood Cell Counts are Decreased After 4 Days of Combined Suspension and Radiation Treatment

**Figure 1:**
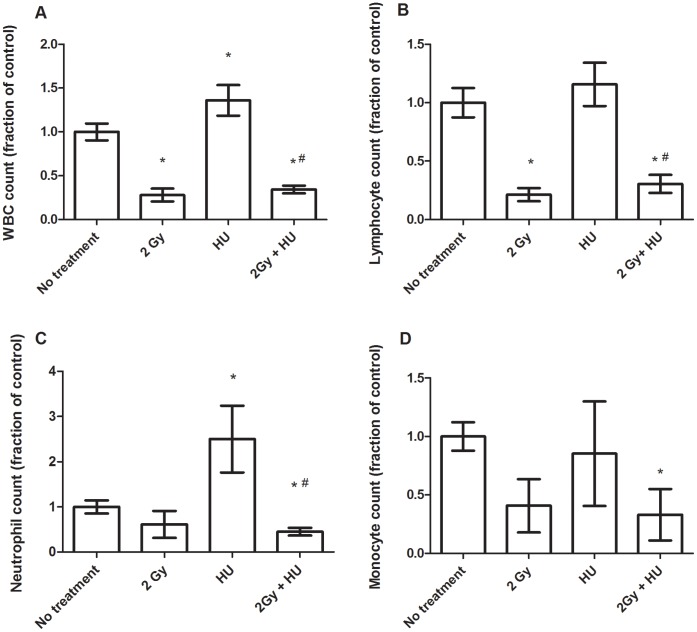
Total blood cell counts are decreased in the irradiated (2 Gy) groups +/− HU treatment WBCs (A) and lymphocytes (B) at 4 days post-proton radiation exposure are significantly decreased, compared to the No treatment group (*). The WBC and lymphocyte results indicated statistically significant differences between the 2 Gy + HU group and the No treatment group (*), as well as between the 2 Gy + HU group and the HU group (#). Average counts +/− SD are expressed as a fraction of the control, n = 3–5. The neutrophil count at 4 days post-proton radiation exposure + HU treatment are significantly decreased, compared to the neutrophil counts observed for the No treatment group (*) and the HU group (#). HU treatment results in a statistically significant increase in neutrophil counts, compared to the No treatment group (*) (C); however, the differences between the neutrophil counts for the 2 Gy group are not statistically significant when compared to the neutrophil counts from the mice in the No treatment group. Monocytes are decreased in the combined treatment group (2 Gy + HU) compared to the No treatment group (*) (D).

At the 4 day time point, the white blood cell (WBC) counts, lymphocyte counts, and neutrophil counts resulted in very similar trends. The WBCs and lymphocytes were decreased in a statistically significant manner in the peripheral blood of animals exposed to 2 Gy with and without HU, compared to the group of animals not suspended and not irradiated (No treatment group, *, Fig. 1A and 1B). The WBCs and lymphocytes were also decreased in a statistically significant manner, when compared to the 2 Gy + HU group to the HU (sham-irradiated) group (#, Fig. 1A & 1B). The absolute neutrophil count was significantly decreased in animals exposed to 2 Gy + HU, compared to the No treatment group (*, Fig. 1C) and the HU (sham-irradiated) group (#, Fig. 1C). HU treatment without radiation exposure resulted in an increased neutrophil count, compared to the No treatment group (*, Fig. 1C). The differences between the results for the neutrophil counts in the 2 Gy treatment group and the No treatment group were not statistically significant. The monocyte count was decreased in a statistically significant manner in the 2 Gy + HU group, compared to the No treatment group, but was not different from the other treatment groups (Fig. 1D). The eosinophil, basophil, and platelet counts did not differ between any of the treatment groups (data not shown).

### Blood Cell Counts are not Changed at the 21 Day Time Point

The differences for all blood cell types analyzed (total WBCs, lymphocytes, neutrophils, monocytes, eosinophils, basophils, and platelets), compared to the No treatment group, were not statistically significant at the 21 day time point (data not shown).

### Splenic Lymphocyte Subtype Populations are Altered After Proton Radiation Exposure and HU Treatment

**Figure 2:**
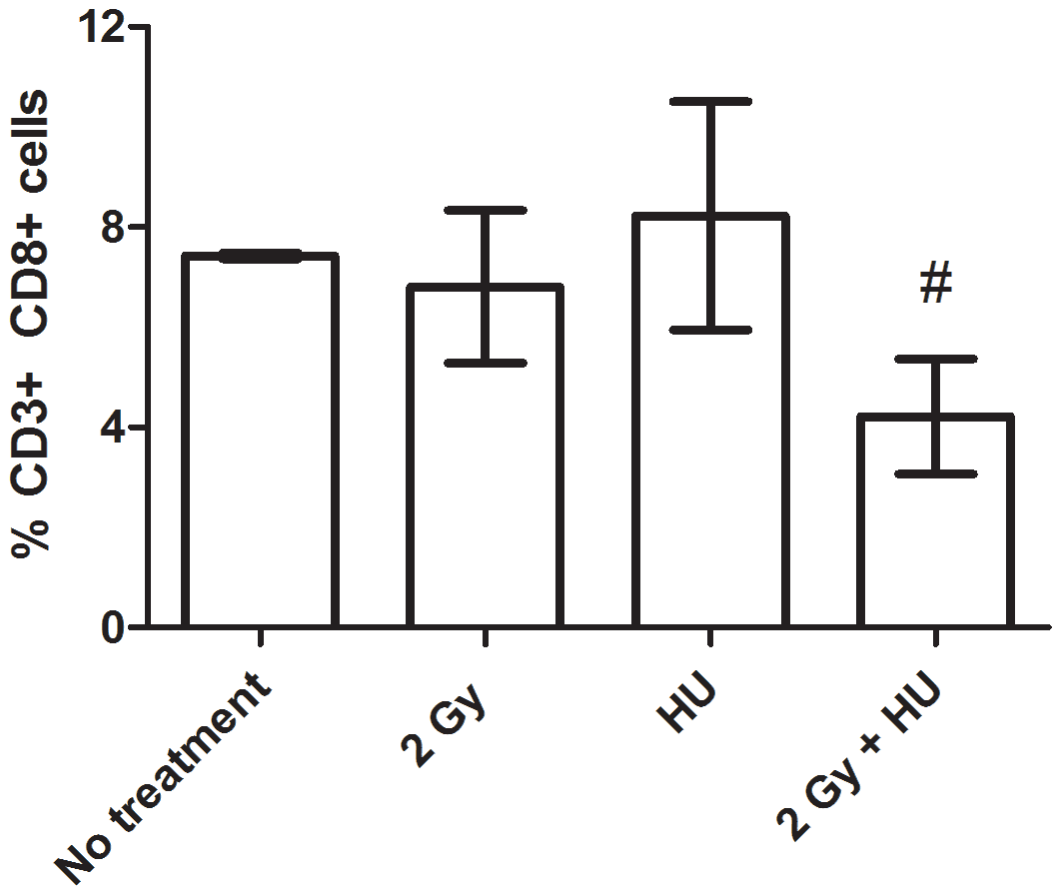
Cytotoxic T cells are decreased in the irradiated and HU animals. At 4 days post-proton irradiation exposure + HU treatment, the population of splenic cytotoxic T cells (expressing CD3+ CD8+ surface markers) is significantly decreased when compared to the HU group, by approximately 50%. The average percent of antigen expressing cells are shown +/− SD, n = 4–6.

At the 4 day time point, splenic lymphocyte subsets and subtypes were determined by fluorescence-activated cell sorting, and there were no statistically significant changes observed in the distribution of lymphocyte subsets (T or B lymphocytes); however, there was a significant decrease in the T lymphocyte subtype, cytotoxic T cells, expressing CD3 and CD8 surface markers, in animals exposed to 2 Gy + HU, compared to animals exposed to HU (#, Fig. 2). In the 2 Gy + HU group, the percentage of T cells expressing CD8+ dropped to ∼50% of the HU group.

**Figure 3:**
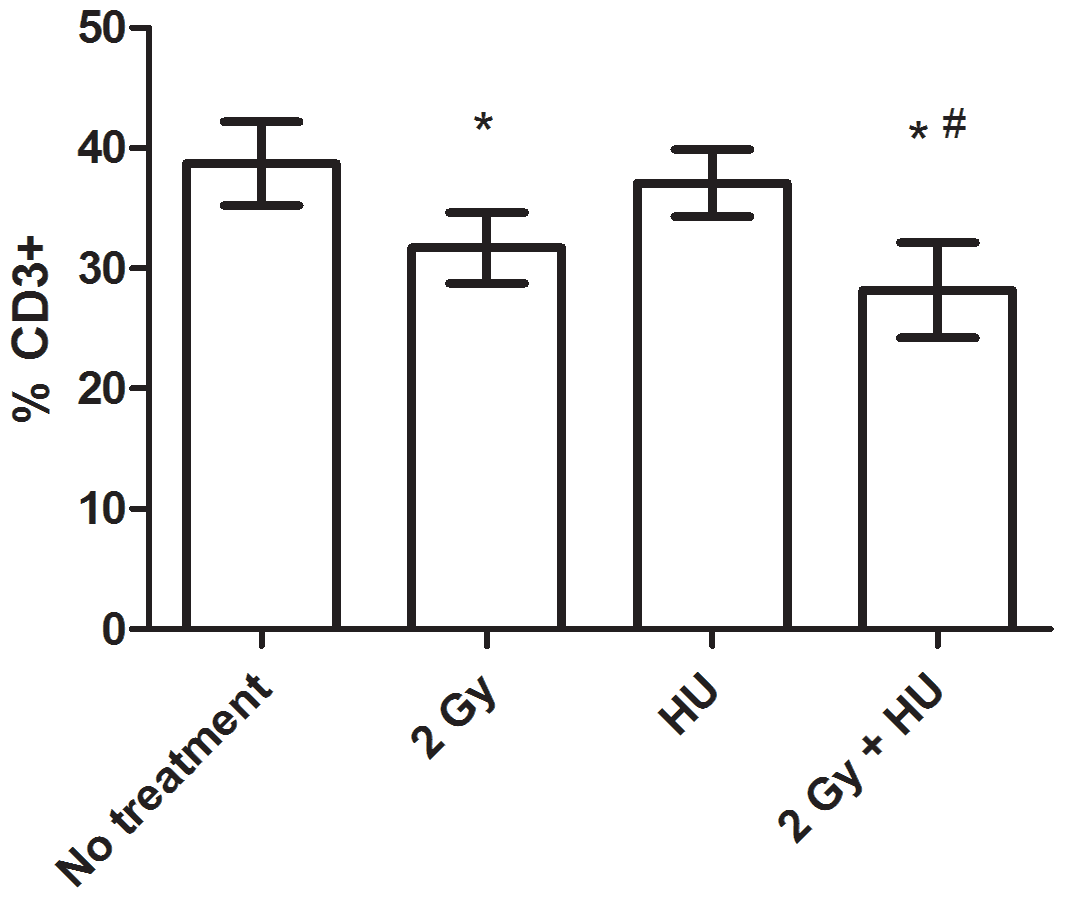
The splenic T lymphocyte population is decreased at 21 days post-proton radiation exposure +/− HU, compared to the No treatment group (*) and/or the HU group (#). The average percentage of CD3+ splenocytes is shown +/− SD, n = 4–6.

At the 21 day time point, the cytotoxic T cell population is similar to all other groups. However, T lymphocytes (CD3+) are significantly decreased in the 2 Gy + HU group, compared to the No treatment group (*) and the HU group (#, Fig. 3). The 2 Gy dose of radiation exposure without HU treatment followed a similar trend, when compared to the No treatment group (*, Fig. 3).

### T Lymphocyte Activation is Suppressed After Proton Irradiation +/− HU

**Figure 4:**
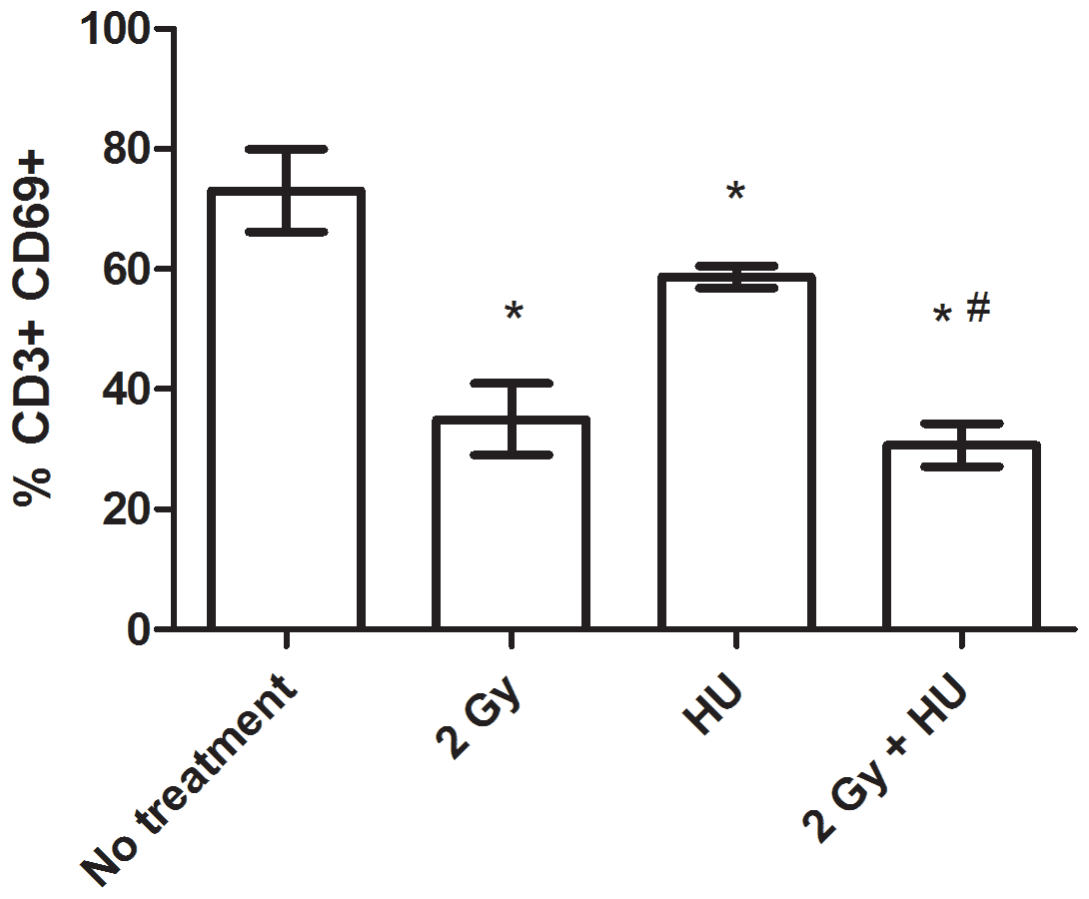
Splenic T lymphocyte activation is suppressed after proton irradiation exposure +/− HU treatment. Compared to the No treatment (*) and HU treatment (#) groups, all groups result in suppressed T cell activation. The percentage of CD3+ CD69+ expressing splenocytes is shown as the average from the group +/− SD, n = 4–5. The 4 and 21 day time point results are comparable, with the 21 day time point results shown here.

Splenic T lymphocytes were isolated and activated *ex vivo*, using a T cell activating cocktail of CD3/CD28/CD137. The very early activation marker, CD69, was detected using flow cytometry (24 hours after activation). All groups result in a lower percentage of CD69+ expression, compared to the No treatment group (*, Fig. 4) and the combined radiation and HU group was also significantly different compared to the HU group (#, Fig. 4). This graph is also representative of results obtained at the 4 day time point, which is comparable to that observed at the 21 day time point (data not shown). The suppression of activation observed after radiation exposure, in the absence and presence of the HU treatment, is ∼30–40% of the No treatment group. HU treatment alone resulted in a 20% depression in T cell activation compared to the No treatment group.

### Isolated T Lymphocytes Demonstrate a Decreased Proliferation Index in Mice Exposed to the Combined Treatment of 2 Gy Proton Irradiation + HU

**Figure 5:**
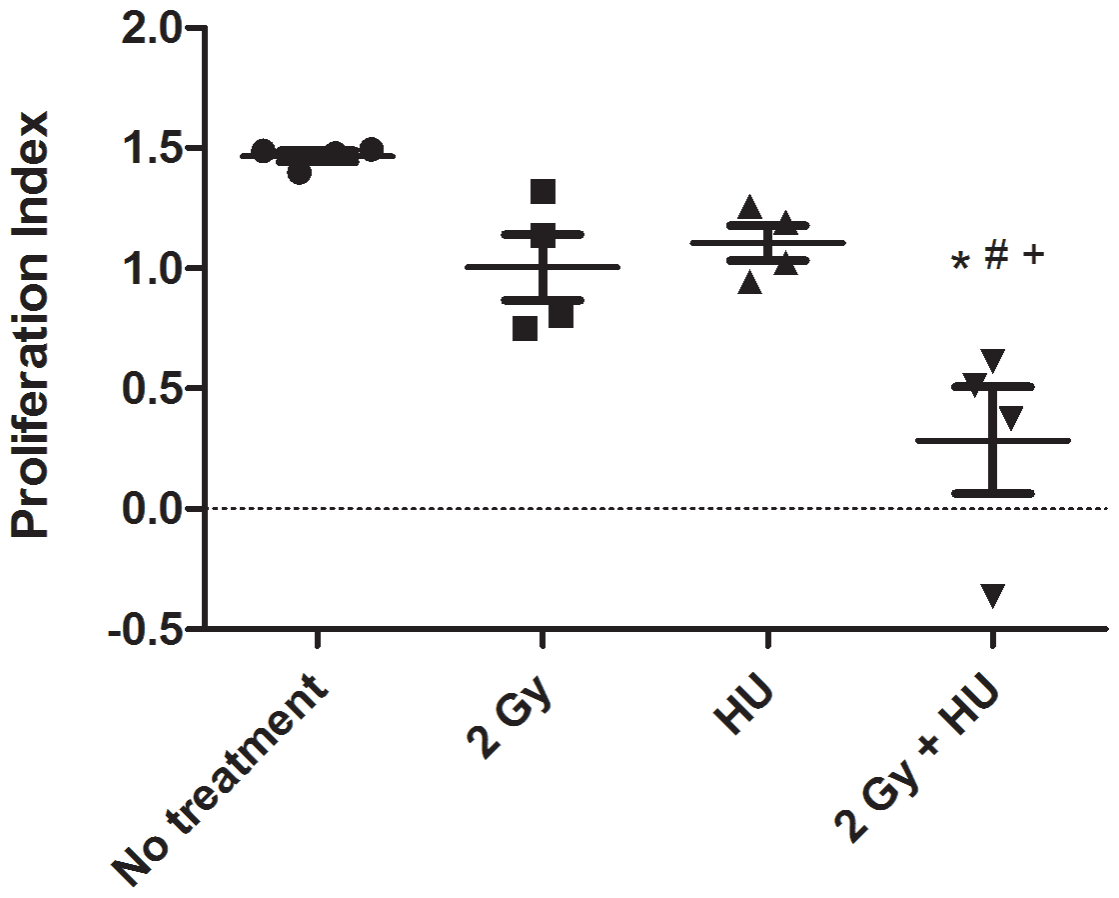
T lymphocytes isolated from animals exposed to proton radiation + HU treatment have a decreased proliferation rate after T cell stimulation. The proliferation index, measured 21 days post-radiation exposure + HU treatment, was significantly decreased when compared to the non-treated (*), 2 Gy (+), and HU (#) groups. The average proliferation index is shown +/− SD, n = 4.

21 days following radiation exposure +/− HU treatment, the non-radioactive kit, MTT Cell Proliferation Assay Kit, was used to measure the cell growth rate of T cells 24 hours after activation. Isolated splenic T lymphocytes from the 2 Gy + HU group have a decreased proliferation index compared to the No treatment (*), the 2 Gy (+), and HU (#) groups (Fig. 5). This assay was not performed in isolated cells from the 4 day time point.

## Discussion

Lymphocyte activation and function are compromised during spaceflight [30]–[32]. This study identified the combined effects of simulated microgravity and proton radiation on circulating hematopoietic cells as well as isolated splenic cells. Absolute cell counts in the peripheral blood were reduced at 4 days post-irradiation and HU treatment, but they were not reduced at the 21 day time point. At the 4 day time point, the HU treatment did not appear to induce a synergistic nor an additive effect in the 2 Gy proton radiation treatment. At both the 4 day and 21 day time points, splenic lymphocyte activation was substantially reduced in both the suspended and non-suspended-irradiated groups, in addition to the HU suspended group without radiation exposure. The irradiated, HU treated group resulted in decreased splenic lymphocyte proliferation 21 days after the combined proton and HU treatment. Taken together, simulated microgravity does not alter the radio-responsiveness of lymphocytes, which corresponds with previous reports using cultured lymphocytes, which utilized clinostatic rotation and gamma-ray radiation [19], [20]. Antiorthostatic suspension has been widely utilized to simulate space flight conditions; the immunological and physiological changes occurring during and after simulated spaceflight have been reviewed [33], [34]. Simulated microgravity, or the HU group studied here, did not result in decreased lymphocyte counts as previously observed in rats upon landing after being flown on the Spacelab shuttle [35]. Contradictory observations have been reported based on physiological stressors that can never be accounted for in ground based studies, including re-entry into the Earth’s atmosphere. Ichiki et al. [36] did not report any changes in the WBC counts of rats euthanized in flight, but such changes were observed upon landing, suggesting that confounding factors are present when evaluating hematological parameters on landing. The neutrophilia observed in animals post-flight (Spacelab shuttle) was observed in the HU treatment group in these studies at only the 4 day time point, and is most likely attributed to stress. In this report, it is plausible that the radiation-induced decrease in peripheral blood cell counts observed at the 4 day time point (post-irradiation) has recovered by day 21 post-radiation. There were no significant changes observed in the CD19+, CD3+, CD3+CD4+ or CD3+CD8+ populations of the HU treated mice in the current study. These results are consistent with the lymphocyte subset phenotyping results in the rats euthanized in flight [36], and it is assumed that, since landing was not part of this study, lymphocyte changes were not observed. Although the circulating blood cell counts appear to have recovered by day 21 post-irradiation in these studies, the functionality of the splenic lymphocytes and the ability to proliferate, is significantly decreased in the animals exposed to the combined microgravity and proton radiation, compared to the untreated control group.

As data from inflight T cell activation studies are limited, it is accepted that T cell activation upon landing after spaceflight is significantly depressed [7], [37]. In agreement with studies using the BIO developed by the National Aeronautics and Space Administration (NASA) [38] (based on clinostat technology), in which suspended lymphocytes were mitogen-activated and activation was depressed by 50% [39], the suspension treatment in the current studies resulted in decreased mitogen activation by 20% and in the presence of the combined treatment of suspension and radiation, by 40%. It is evident that simulating the spaceflight environment (combined radiation and microgravity exposure) on Earth has limitations, but the results reported here clearly indicate lymphocyte dysfunction which may impact the health of crew members.

This is the first demonstration in a ground-based animal model system that radiation exposure, with or without hindlimb suspension, as well as hindlimb suspension by itself, leads to a lack of activation of splenic T cells. It is known that T cells have extremely important signaling functions related to the coordination of immune system response(s) to invading pathogens or other traumatic insults to an organism (e.g., exposure to radiation). The full significance of the lack of T cell activation cannot be determined from the results presented here, but results from other investigators suggest that our results may have great significance for the ability of animals and people to resist the development of infections during space travel and after exposure to significant doses of radiation. It has been previously shown that in simulated microgravity (hindlimb suspended) conditions, animals have an impaired ability to deal with pathogens [4], [40]–[43]. It has also been observed that mice exposed to space (proton) radiation either with or without hindlimb suspension or hindlimb suspension by itself, lead to mortality when they are challenged with non-toxic doses of bacteria (*Pseudomonas aeruginosa* or *Klebsiella pneumoniae*); in these experiments, exposure of mice to both proton radiation and hindlimb suspension leads to very high levels of mortality (Drew Weissman, M.D., Ph.D., M. Li and colleagues, unpublished data). In these studies, a dose of 2 Gy proton radiation was used; a 2 Gy dose of radiation is a normal dose of radiation used in clinical radiotherapy procedures and is a dose of radiation that astronauts can receive during exposure to solar particle event (SPE) radiation [44]. People exposed to radiation at significant doses are known to develop a higher than normal rate of infections, as are astronauts. Infections of the skin, eyes and respiratory tract have been reported 13 times in the Apollo and 8 times in Skylab missions; those spacecrafts were equipped with numerous antibiotics (Tetracycline, Ampicillin, and Neosporin) to serve as countermeasures for these infections [45], [46].

### Conclusion

Thus far, astronauts have not been exposed to the high doses of radiation that can be received by exposure to SPE radiation [47], but exposure to SPE radiation will be considerably more likely for astronauts during the exploration class missions (with the necessary extended times in space travel) planned by NASA and other space agencies for the future. Although there may be other potential explanations for the high infection rates in astronauts and others exposed to radiation, our results suggest that the lack of T cell activation could be at least partially responsible for the enhanced susceptibility in these populations to the development of infections. It is worth noting that radiation and hindlimb suspension both have long-lasting effects on T cell activation in our studies, with the lack of T cell activation from exposure to either radiation or hindlimb suspension lasting for at least 21 days, which is the maximum period of time post-exposure that T cell activation was evaluated in the studies reported here. Such a long-lasting effect could have major consequences during and after space flight and in irradiated populations, which could include cancer patients treated with 2 Gy doses of radiation in the clinic.
